# Spinal Surgery

**Published:** 1895-01-12

**Authors:** 


### SPINAL SURGERY.

**Tumour.**—Ferrier[1] and Watson Cheyne report a case of fibro-myxoma of the spine in a man aged 25. Eight years before operation the symptoms set in with pain and tenderness in the region of lower ribs on the right side. For two years afterwards he was able to continue at work as a fisherman, then he began to complain of difficulty in raising the right foot in walking, and of tenderness in the sole on standing. A little later the left leg became similarly affected. During the next four and a-half years these symptoms increased in severity, and then "cramps in the legs," and frequent micturition set in, and two months later the bladder became paralysed. When seen by Ferrier previous to operation the patient was obliged to maintain a sitting posture with thighs flexed on pelvis and knees bent. Change from this position caused violent extensor spasms of the legs and pain shooting up the back, especially on the right side. He complained of pain in the region of the last three ribs on the right side, occasionally shooting forwards to the epigastrium and backwards to the spine. There was paralysis of the lower limbs and bladder. The knee-jerks were exaggerated and ankle clonus was present. Sensibility was wholly lost up to the level of the second lumbar spine, and diminished up to the level of the umbilicus. Deep pressure caused pain over the spines from the seventh to tenth dorsal vertebræ. From the symptoms and a comparison with former cases Ferrier diagnosed the presence of a tumour probably intradural. Laminectomy was performed by Watson Cheyne, and a soft intradural fibromyxoma, which extended from the sixth dorsal lamina down to the eleventh, was removed without injury to the cord or nerve. The patient sank and died one and a half days after operation.

**Tuberculosis.**—Roberts[2] records a case of primary tuberculosis of the laminæ and spinous processes—a rare condition—in a girl with paraplegia. There was a distinct swelling in the middle of the dorsal region. It was most marked on each side of the median line, and there was a hollow over the spinous processes. From the nature of the deformity tuberculosis of the laminæ was suspected. Laminectomy was performed, but during the operation the child became very weak, and shortly afterwards died.

Beck[3] performed laminectomy in a case of tubercular spondylitis. The ninth and tenth dorsal vertebræ were found to be affected, and the diseased bone removed. The patient made an excellent recovery, a large gluteal abscess healing up. Beck recommends the treatment of psoas and peritoneal abscesses by puncture and injection of iodoform glycerine, and uses a double barrelled canula, which allows of the forcible irrigation of the cavity, and the washing out of caseous fragments.

Burr[4] discusses the cause of the nervous symptoms in Pott's Disease. He considers that in the majority of cases these do not arise from pressure due to the bony deformity, but from inflammatory changes in the dura. When the osteitis has reached the posterior surface of the vertebral body, ulceration of the posterial common ligament takes place, and then pachymeningitis follows. Soon this causes pressure on the cord, and sclerosis results, the peripheral nerves becoming affected in the same way as they pass through the dura.

**Injuries to the Spine.**—Mr. Bennett[5] insists on the necessity of treating every injury to the spine with great caution, as "serious injury inside the vertebral column is not necessarily associated in the first instance with any gross evidence of injury to the spinal cord." Any inability to pass urine, or feeling of tingling, creeping, or "pins and needles" after an injury to the spine should be looked upon as warnings, even although the patient seems otherwise quite well, and although they quickly pass off. Neglect of them may lead to the nature of the case being overlooked till secondary changes in the cord cause marked symptoms. He records several cases in which at first the injury to the spine seemed to have caused little hurt to the patient, but in which paralysis developed after an interval of hours or days. Considerable hæmorrhage into the vertebral canal may occasion at first very little in the way of symptoms. Newton[6] reports a case of fracture and dislocation of the second lumbar vertebra in which the cauda equina was injured. He performed laminectomy, but the patient died six days later. Gordinier[7] records a case diagnosed as injury to the cauda equina from hæmorrhage after a fall. Marked improvement had taken place in the paralysis at the end of five months. Baumüller[8] has successfully reduced a subluxation of the second lumbar vertebra. A year afterwards slight paresis of the right tibial region was all that remained of the nervous symptoms, which were well marked at time of injury. Roberts[9] reports four cases of laminectomy for old spinal fracture. Results were very unsatisfactory, and lead him to doubt the value of operation in old cases. He considers early operation in spinal fracture as essential as in cranial fracture. Laminectomy was performed unsuccessfully by Giles[10] in recent fracture of the seventh and eighth dorsal vertebra in an adult. Patient died twenty-four days afterwards. Kiär[11] describes an interesting case of a stab in the spinal cord at the level of the first dorsal vertebra, recovery taking place. "Railway spine" is dealt with in a paper by Blum.[12] He does not consider it due to concussion of the cord, as he doubts the possibility of such a condition, but classes it as a form of hysteria or neurasthenia.

**Spina Bifida.**—At the International Congress, in Rome, Mayo Robson[13] read a paper on this subject, and gave a synopsis of twenty operations with fifteen recoveries. He advocates treatment by a plastic operation. The greatest care is necessary as primary union is essential for a good result. He considers operation inadvisable where there is marked paraplegia, hydrocephalus, or marasmus, and in cases where the tumour is small and has a firm covering of integuments.

**Coccydynia.**—Barwell,[14] who has seen twenty-three cases, deals with this subject in an interesting paper. Coccydynia is much more frequent in women than in men. While this may be partly due to greater nervous susceptibility and to uterine conditions, it is probably accounted for by the greater width between the tuberosities of the ischium rendering the coccyx more liable to injury by falls, blows, or pressure. It may occur at all ages. The causes are various. As a rule there has been a fall, blow, or prolonged pressure on the coccyx. Sometimes, however, there is merely a history of long ill-health with continued over-fatigue, at times associated with prolonged sitting on a hard seat. Neglected fissure of the anus and blind internal fistula have been the cause in some cases. In only one of Barwell's cases did the patient give childbirth as the cause, and in that case the proof was inconclusive, as the pain did not come on till seventeen months afterwards. Mr. Barwell believes that apart from neuralgia in the majority of cases the condition present is one of myalgia and spasm of the muscles attached to the coccyx. He divides the cases into two classes—one in which the muscles passing forwards from the coccyx, *i.e.*, the perineal muscles are involved, and the other in which the muscles passing backwards, the glutei, are affected. In the first class the pain, as a rule, is most marked during defæcation, passage of flatus, and micturition; the sitting posture, though causing uneasiness, does not produce severe pain when the affected parts are not pressed on, and short walks may be taken without discomfort. The sphincter ani is tight, and even slight dilatation causes great pain. Pressure backwards on the tip of the coccyx from the rectum is very painful, the levator ani is tender, and pressure on it may set up spasm. In the other class defæcation, &c., cause no increase of pain, but sitting in any position is painful, while rising from a chair; stooping and walking cause great discomfort. Rectal examination does not elicit the symptoms enumerated above. Mixed cases occur in which both sets of symptoms are present. Medicinal treatment gives only temporary relief, and tenatomy is, as a rule, necessary. In the first class of cases Barwell divides the levator ani, coccygeus, and sphincter ani from the coccyx. In the other class he divide the fibres of the glutei muscle attached to the coccyx, and if necessary the lowest fibres of the lumbar fascia. In mixed cases he does both operations. He has operated on seventeen cases with recovery in all.

[1] Lancet, March 24, 1894, p. 739. [2] Annals of Surgery, March, 1894, p. 597. [3] Amer. Med. and Surg. Bulletin, Feb. 1, 1894, p. 145. [4] Med. Chron., March, 1894, p. 408. [5] Clinical Journal, April 11, 1894, p. 376. [6] Amer. Jour. Med. Sc., April, 1894, p. 423. [7] Lancet, May 26, 1894, p. 1316. [8] Brit. Med. Journ., May 26, 1894, Ep. 82. [9] Brit. Med. Journ., April 14, 1894. [10] Brit. Med. Journ., May 19, 1894, Ep. 78. [11] Lancet, March 17, 1894, p. 692. [12] Med. Chron., March, 1894, p. 337. [13] Lancet, April 14, 1894, p. 962. [14] Medical Week, March 30, 1894, p. 150.

